# The sweet taste of death: glucose triggers apoptosis during yeast chronological aging

**DOI:** 10.18632/aging.100223

**Published:** 2010-11-02

**Authors:** Christoph Ruckenstuhl, Didac Carmona-Gutierrez, Frank Madeo

**Affiliations:** Institute of Molecular Biosciences, University of Graz, 8010 Graz, Austria

**Keywords:** autophagy, chronological lifespan, longevity, superoxide, ROS, growth signaling, group selection theory

## Abstract

As time goes by, a postmitotic cell ages following a degeneration process ultimately ending in cell death. This phenomenon is evolutionary conserved and present in unicellular eukaryotes as well, making the yeast chronological aging system an appreciated model. Here, single cells die in a programmed fashion (both by apoptosis and necrosis) for the benefit of the whole population. Besides its meaning for aging and cell death research, age-induced programmed cell death represents the first experimental proof for the so-called group selection theory: Apoptotic genes became selected during evolution because of the benefits they might render to the whole cell culture and not to the individual cell.

Many anti-aging stimuli have been discovered in the yeast chronological aging system and have afterwards been confirmed in higher cells or organisms. New work from the Burhans group (this issue) now demonstrates that glucose signaling has a progeriatric effect on chronologically aged yeast cells: Glucose administration results in a diminished efficacy of cells to enter quiescence, finally causing superoxide-mediated replication stress and apoptosis.

More than a decade ago, our group discovered that dying yeasts do not succumb in an unregulated manner but rather follow a highly orchestrated molecular choreography, which resembles mammalian apoptosis in both morphological and molecular terms. On the one hand, morphological apoptotic markers, such as phosphatidylserine externalization, DNA degradation, chromatin condensation, and the generation of reactive oxygen species (ROS), accompany yeast apoptosis [1,2]. On the other hand, crucial molecular regulators of apoptosis in yeast, including core executors like a caspase [3], the apoptosis-inducing factor [4], endonuclease G [5] or the serine protease OMI [6], as well as pivotal inhibitors like the AAA-ATPase *CDC48* (p97/VCP) [7] or the IAP (inhibitor of apoptosis protein) *BIR1* [8], have been identified by different groups. Moreover, yeast apoptosis has been causally linked to complex metabolic scenarios such as the Warburg effect [9] or lipotoxicity, a form of cellular demise resulting from lipid overload [10]. Other „classical” apoptosis features connected to dying yeasts are deregulated mitochondrial fission and fusion, cytochrome c release, perturbations of the actin or tubulin cytoskeleton, and epigenetic modifications of the chromatin [11-15].

Research in this area has also provided a teleological explanation for regulated yeast cell death, which a priori should be counterproductive for a unicellular organism, by proving its fundamental role in several physiological scenarios, among others viral infection, meiosis, mating and aging [16-18]. In these scenarios, the death of damaged individual cells yields a selective advantage for the yeast population as a whole [17-19], facilitating the spreading of the clone. This is also the case during chronological aging of yeast cells, a model invented and developed by V. D. Longo in 1996 [20] and defined by the decline of surviving cells in the postmitotic stationary phase, thus simulating the aging of the mostly postmitotic cells of higher organisms. Here, programmed death of old, damaged yeast cells (both by apoptosis and necrosis [17,18,21]) favors the long-term survival of the population. For instance, a strain devoid of the apoptotic machinery or overexpressing superoxide dismutase (and therefore with diminished levels of superoxide) shows an initial advantage in a direct over-time competition assay with a wild type strain; however, it gets finally outcompeted by the wild type strain because it accumulates damaged or unfit cells [17,18]. Programmed cell death seems to clean the population over time, suggesting that aging in yeast (and possibly in higher organisms) may be programmed, since single cells sacrifice themselves for the benefit of the group. In fact, these data may be regarded as the first experimental proof for the so called „group selection theory" as proposed by A. Wallace, in which it is suggested that alleles can become selected because of the benefits they might render to the group, not to the individual [17,22].

Besides such philosophical considerations, the yeast chronological aging system (Figure 1) has led to the discovery of aging mechanisms and anti-aging drugs that have subsequently been confirmed in higher organisms [23]. Examples include branched chain amino acids (BCAA), first found to extend yeast chronological lifespan (CLS) and then confirmed as regulators in mice [24,25] or spermidine, first detected in yeast as an antiaging compound upon external administration and afterwards shown to also prolong life of flies, worms, human immune cells, and, possibly mice [21,26]. CLS extension by rapamycin, was first shown in budding yeast and meanwhile shown to promote longevity in higher eukaryotes (i.e. flies and mice) as well [27-29]. Furthermore, FCCP a mitochondrial uncoupler extended CLS of yeast as well as lifespan of worms [30,31].

**Figure 1.**
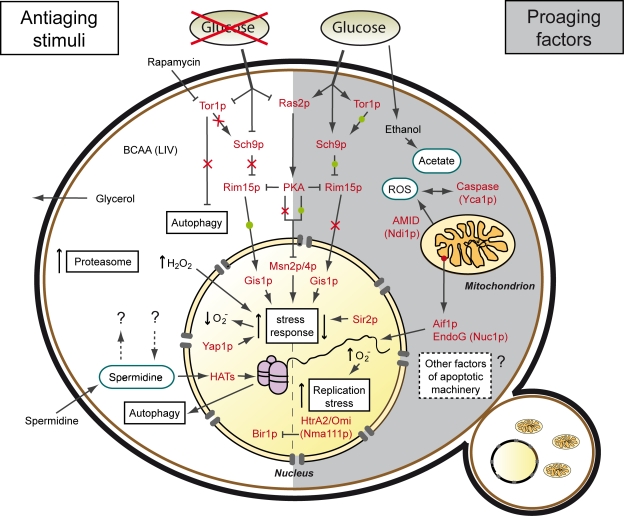
Stimuli and factors involved in yeast chronological aging. The process of chronological aging ultimately relies on a cell's decision to stall or promote its growth in a given scenario. If growth is inhibited, for instance due to low nutrient availability (caloric restriction), the cell enters a state of low metabolic activity (quiescence), thus arresting the aging process (antiaging). If nutrients are available the cell activates growth progression, elevates metabolic rates, promotes its reproduction and progressively ages (non-quiescence or senescence), eventually culminating in its demise (proaging). Consequently, glucose availability has a major impact on longevity, specifically via the Tor-Sch9p and Ras2-PKA pathways, which require the serine-threonine kinase Rim15p and the transcription factors Gis1p, Msn2p and Msn4p to regulate expression of stress response genes. While longevity is promoted upon low glucose availability (↓glucose → ↓Tor/Sch9p/Ras2 → ↑stress response), it is shortened when glucose availability is high (↑glucose → ↑Tor/Sch9p/Ras2 → ↓stress response). Stress response is also regulated via other factors like the transcription factor Yap1p or the histone deacetylase Sir2. The Tor1p kinase is also involved in the regulation of autophagy, a self-recycling pathway under nutrient starvation. Upregulation of autophagy may occur via inhibition of the Tor1p kinase (for instance with rapamycin) or elevation of intracellular spermidine levels (by external supplementation or internal regulation mechanisms still to be discerned). Spermidine, which induces autophagy via inhibition of histone acetyl transferases (HATs) and resulting histone hypoacetylation, could also regulate longevity via autophagy-independent alternative pathways. In addition, chronological lifespan is extended by increased availability of branched side chain amino acids (BCAA; leucine, isoleucine and valine) and a rise in synthesis and release of glycerol, which in turn is inhibited by sustained growth signalling (see text). On the other hand, under conditions where chronological aging is promoted ethanol is metabolized to acetate, which acts as a proaging trigger, in part by influencing internal and external pH as well as TOR signaling. Additionally, growth signaling leads to cell cycle progression and replication stress. Chronological aging-induced cell death has been shown to be regulated by a number of mitochondrial, nuclear and cytosolic lethal effectors and might also involve further factors associated with the yeast apoptotic machinery.

The causative role for ROS as a driving force of the aging process serves as the unifying feature of the CLS model. Fabrizio et al. presented evidence, that the superoxide-induced demise of yeast cells during chronological aging provides the population with nutrients and could favor either spontaneous or specific mutations leading to the so-called adaptive regrowth [17]. Adaptive regrowth occurs in late phases of chronological aging experiments where already 90-99% of the population is dead and describes the growth of better adapted mutants, which is facilitated by nutrients released from dead cells. Interestingly, long-lived mutants (like *sch9* or *ras2* deletion strains) do not show adaptive regrowth, possibly due to the diminished superoxide production of these strains [17].

In this issue of AGING Weinberger et al. present further evidence supporting the idea of superoxide anions acting as signal molecules that determine yeast CLS [32]. While activation of the major growth signaling pathways (TOR, AKT/Sch9p in *Saccharomyces cerevisiae* and RAS) by several triggers (e.g. high glucose levels) increases superoxide levels, inactivation of these signaling pathways by caloric restriction (CR) or gene deletions (*SCH9* or *TOR1*) diminishes superoxide levels. CR-effects are in part dependent on Rim15p [33]; in addition, Weinberger et al. could show that Rim15p-independent effects are mediated by H_2_O_2_-induced expression of superoxide dismutases, which results in reduced superoxide levels [32,34]. Furthermore, high superoxide levels correlate with the inability to cleanly arrest in G0/G1 growth phase shifting the arrest rather to the S phase, which in turn induces replication stress and hinders quiescence [32]. Quiescence is a prerequisite for postmitotic cell survival [19] and replication stress a known inducer of apoptosis in *S. cerevisiae* [35].

One of the key triggers of growth signaling used by Weinberger et al. is elevated glucose. Interestingly, exposure to pure glucose in aqueous solution has been shown to induce cell death in stationary yeast cultures [36]. The occurring cell death, which is of apoptotic nature, can be prevented by inhibiting ROS production through addition of ascorbic acid [37]. Intriguingly, Granot and colleagues could also demonstrate that glucose induced early, pre-budding growth events (culminating in faster budding upon transfer to rich media) [36,38], suggesting that apoptotic death can even occur when budding is in preparation, but not visible yet. Given that these conditions (stationary yeast in aqueous glucose solution) possibly resemble later phases of chronological aging experiments with glucose excess, the superoxide-based mechanism proposed by Weinberger et al. [32] might underlie the effects observed by Granot et al. [37].

The understanding of the proposed superoxide signal cascade will require clarifying the molecular connection between growth signaling pathways and the generation of superoxide. Notably, superoxide is a byproduct naturally occurring during respiration and other enzymatic reactions [39]. Mitochondria, well-established executive organelles in both apoptosis and aging [40-42], represent the main sites for superoxide leakage, in particular complex I (in yeast consisting only of NADH-ubiquinone oxidoreductases) and complex III of the respiratory chain [43]. The increase in superoxide levels accompanying stable growth signaling could, thus, be an unavoidable byproduct of enzymatic reactions (i.e. during limited respiration). However, it is also conceivable that under such circumstances superoxide might be produced specifically as a signaling molecule by appropriate enzymes (e.g. NAD(P)H oxidases).

In budding yeast administration of glucose leads to drastically diminished respiratory activity due to a very effective glucose repression circuit [44,45]. Albeit, NADH (that is generated during the conversion of sugar into biomass) and possibly other upstream metabolites for reductive potential and use in respiration will still lead to basic respiratory activity and could derive in a saturation of the electron transport capacity under conditions, where low expression of respiratory chain components limit respiration. While overall ROS production (via respiration) should remain rather low, specific superoxide generation could occur. Interestingly, the group around G. S. Schadel has previously published that TOR signaling and Sch9p kinase are downstream effectors of respiration and ROS production [46]. Limiting TOR signaling during glucose availability results in enhanced O_2_ consumption (without rising ATP levels) and elevated expression of respiratory complexes but diminished ROS production during early time points [46]. These observations might support the above mentioned concept of specific superoxide generation under conditions of glucose repression during early growth phases in an aerobic environment.

Under anaerobic conditions glycolytically generated NADH cannot be deployed to respiration and thus is re-oxidized by generating glycerol, a reaction that consumes reductive potential by NADH oxidation [47,48]. Intriguingly, Wei et al. could show that the *sch9* long-lived deletion mutant, which generates less superoxide, produces and exports glycerol under aerobic growth conditions when glucose or ethanol are available, thus generating a CR-like environment [49]. In this case - taking together the aforementioned considerations - NADH would be redirected from being used in respiration and could account, in part, for the lowered ROS accumulation found in this mutant strain. This is in line with the observed altered respiration phenotype of *sch9* mutants, whose respiration is higher during early [46] and lower during later growth phases [49] as compared to the wild type: while during early growth (high levels of glucose) an overload of electrons in the respiratory chain would be circumvented, thus making respiration more efficient in comparison to the wild type, overall NADH availability for respiration would be reduced in later growth phases. It is tempting to speculate that glycerol production and release by long-lived mutant yeast strains reflects a general concept also important for higher organisms. In that sense it is remarkable that worms fed on glucose present reduced longevity likely due to a down-regulation of a specific aquaporin glycerol-tansport channel [50].

Of note, in a different yeast model with continuous high growth rates (grown as colonies on agar plates) cells can be protected from ROS-induced apoptosis in early growth phases by eliminating the onset of respiration either via higher glucose levels or via deletions resulting in loss of respiration competence [9]. Additionally, abrupt induction of respiration in highly proliferative cells (i.e. by shifting exponentially growing yeast cells on respiratory media) results in ROS-mediated apoptosis that can be avoided by addition of glutathione [9]. These results emphasize the importance of different growth models and hint to different roles for ROS during different growth phases and scenarios.

Besides its actual production, both the cellular localization of superoxide and the mechanism signaling an S phase rather than an G0/G1 phase arrest ultimately driving cells into replication stress are of great interest. ROS have been known for a long time to induce cell cycle alterations; however, focus was directed towards secondary effects mediating cellular damage [51]. Intriguingly, recent publications ascribe ROS cell cycle-associated signaling attributes: for instance, Hole et al. [52] recently described that Ras-induced ROS generation by an NADPH oxidase promotes growth factor-independent proliferation in human CD34+ hematopoietic progenitor cells and Cova et al. [53] evidenced that alteration of cell cycle in Amyotrophic Lateral Sclerosis disease is linked to mutated Superoxide dismutase 1.

The results by Weinberger et al. suggest that different ROS trigger specific effects, making it particularly important to accurately distinguish them and their downstream activity (either as damaging agent or signal molecule). At the same time, Weinberger et al. also provide evidence that acetic acid production (and/or resulting pH alterations), which, in some settings is able to restrict yeast CLS [54-56], can lead to sustained growth signaling. This observation could have theoretical and practical implications for a variety of human diseases [55]. For example, blood pH, which declines in humans with ongoing aging due to renal insufficiencies, can be influenced by our diet. In fact, modern diets could lead to a low-grade systemic metabolic acidosis [57,58].

Metabolic homeostasis, which can be regulated by autophagy during chronological aging, is a key component of longevity. Intriguingly, main signaling pathways involved in autophagy (e.g. TOR) were shown by Weinberger et al. to modulate superoxide levels [32]. Consistently, lifespan extension by diminished TOR/Sch9p signaling leads to decreased superoxide levels [32,59] and elevated autophagy [24]. Our group could demonstrate that addition of spermidine (a natural occurring polyamine) drastically extends CLS dependent on induction of autophagy and accompanied by elevated fractions of quiescent cells. The longevity-promoting effects of spermidine addition were absent in autophagy deficient cells in a pH-independent manner [21]. Furthermore, cultivation of yeast in the presence of rapamycin elongates lifespan in an autophagy-dependent manner as shown by Alvers et al. [60]. In these experiments, rapamycin was added right after inoculation of yeast cultures emphasizing that early onset of autophagic events might already pave the impact on longevity.

It will be interesting to see if autophagy and superoxide effects are strictly parallel, but cooperative events in promoting longevity, or if superoxides have a direct effect on the regulation and/or occurrence of autophagy and vice versa. Indeed, a number of works have shown that ROS stress is an important inducer and regulator of autophagy [61]. For instance, Atg4p (an essential protease of the autophagic machinery) is a direct target for oxidation by H_2_O_2_ [62].

After the pioneering work of Longo and colleagues [17,20,59] on the role of superoxide in yeast CLS, Weinberger et al. now reinforces the importance of superoxide and provides exciting new aspects of its role in determining CLS [32]. It now needs to be clarified how it is interconnected to other known parts of the regulatory picture determining (yeast) lifespan. Given that high glucose uptake also negatively influences lifespan of higher organisms like *Caenorhabditis elegans* [31,50] accompanied by elevated superoxide levels [31], these results may well open doors to gain regulatory insights into how our diet influences our lifespan and our health.
